# Prevalence and Predictors of Major Depression in HIV-Infected Patients on Antiretroviral Therapy in Bamenda, a Semi-Urban Center in Cameroon

**DOI:** 10.1371/journal.pone.0041699

**Published:** 2012-07-31

**Authors:** Bradley N. Gaynes, Brian W. Pence, Julius Atashili, Julie O’Donnell, Dmitry Kats, Peter M. Ndumbe

**Affiliations:** 1 Department of Psychiatry, University of North Carolina School of Medicine, Chapel Hill, North Carolina, United States of America; 2 Department of Community and Family Medicine, Duke Global Health Institute, and Center for Health Policy and Inequalities Research, Duke University, Durham, North Carolina, United States of America; 3 Department of Public Health and Hygiene, University of Buea, Buea, Cameroon; 4 Department of Epidemiology, Gillings School of Global Public Health, University of North Carolina, Chapel Hill, North Carolina, United States of America; 5 Department of Biomedical Sciences, University of Buea, and Department of Microbiology and Immunology, University of Yaounde I, Buea, Cameroon; Institut National de la Santé et de la Recherche Médicale, France

## Abstract

Recent blue-ribbon panel reports have concluded that HIV treatment programs in less wealthy countries must integrate mental health identification and treatment into normal HIV clinical care and that research on mental health and HIV in these settings should be a high priority. We assessed the epidemiology of depression in HIV patients on antiretroviral therapy in a small urban setting in Cameroon by administering a structured interview for depression to 400 patients consecutively attending the Bamenda Regional Hospital AIDS Treatment Center. One in five participants met lifetime criteria for MDD, and 7% had MDD within the prior year. Only 33% had ever spoken with a health professional about depression, and 12% reported ever having received depression treatment that was helpful or effective. Over 2/3 with past-year MDD had severe or very severe episodes. The number of prior depressive episodes and the number of HIV symptoms were the strongest predictors of past-year MDD. The prevalence of MDD in Cameroon is as high as that of other HIV-associated conditions, such as tuberculosis and Hepatitis B virus, whose care is incorporated into World Health Organization guidelines. The management of depression needs to be incorporated in HIV-care guidelines in Cameroon and other similar settings.

## Introduction

More than 25 million people have died from HIV/AIDS, most of them in sub-Saharan Africa (SSA) where HIV/AIDS is the leading cause of mortality. [1] In 2010, SSA accounted for approximately two-thirds of global cases. [1] Despite recent global initiatives to improve the availability of and access to antiretroviral therapy (ART) in SSA, the continued high adult mortality due to HIV/AIDS in this region results in significant social and economic consequences.

Cameroon, located in central Africa, has an estimated 550,000 persons living with HIV and an HIV prevalence rate of 5.5%. [2] In a number of ways, Cameroon’s experience with HIV is representative of many SSA countries. The HIV prevalence in Cameroon approximates the mean HIV prevalence in SSA (6.8%). [2] Further, as with the majority of low- and middle-income countries, and the majority of SSA nations, 20–39% percent of those eligible for ART receives the medications. [1] Cameroon is also one of 22 priority countries identified in the World Health Organization’s “Global Plan” as a key target because of the high estimated numbers of pregnant women living with HIV. [3]

Mental disorders, especially depression, are common in HIV-infected persons globally. The HIV/AIDS Costs and Services Utilization Study (HCSUS) study, a U.S. national study of HIV-infected individuals, found that nearly half (48%) of participants had a probable mental disorder. [4] The major mood and anxiety disorders are five to ten times more prevalent in HIV-positive individuals than in the general U.S. population, [5] with a similar increased risk found in SSA settings. [6] The most common psychiatric diagnoses among HIV-positive individuals are mood and anxiety disorders, particularly MDD and other depressive disorders. [5], [6], [7], [8], [9], [10] Similar data are limited for persons with HIV in SSA generally and, to the best of our knowledge, are lacking for Cameroon.

Depression is associated with worse HIV-related outcomes. In individuals with HIV/AIDS, mental illness (MI) in general and depression in particular, have been consistently associated with negative HIV-related behaviors, particularly poor ART adherence, a critical consideration in HIV care where ART plays a central role in suppressing virus and protecting the immune system. [11], [12], [13], [14], [15], [16], [17] A recent meta-analysis of 95 studies encompassing 35,029 participants confirmed the consistent association of depression with poor ART adherence low-resource and high-resource settings. [18] Additionally, depression predicts a higher likelihood of engaging in unsafe needle-sharing and sexual behaviors that risk secondary transmission of HIV infection. [19], [20] Depression has also been associated with poorer physical health, [21] decreased quality of life, [22] and AIDS-related mortality. [23] Thus, identifying those at risk of a depressive illness can highlight patients especially in need of active follow-up.

Recent prominent blue-ribbon panel reports have concluded that HIV treatment programs in less wealthy countries must integrate mental health identification and treatment into normal HIV clinical care and that research on mental health and HIV should be a high priority, especially in less wealthy countries. [24] Cameroon is one such country, and no prior studies of depression prevalence in HIV patients in Cameroon exist.

Accordingly, we sought:

- To determine the prevalence of clinically relevant depressive illness in HIV patients in a small urban setting in Cameroon, and
- To describe the severity of MDD in the patient population and to identify sociodemographic and clinical variables associated with a greater risk of depressive illness.

## Methods

### Ethical Approvals

This research was approved by the Institutional Review Boards of the University of North Carolina, Duke University, and the Cameroon National Ethics Committee.

### Study Design/Population

Bamenda, a city in northwestern Cameroon and capital of the North West Province, has a population of 269,530, [25] making it the country’s third most populated center. We conducted a cross-sectional study of HIV-infected patients on antiretroviral therapy (ART) attending the Bamenda Regional Hospital AIDS Treatment Center (BRHATC). This health facility is dedicated to the care of HIV-infected patients, the vast majority of whom are on ART. The center provides care to over 1,000 patients annually. Between May 2010 and October 2010, attendees of the BRHATC were informed of the study through thrice-weekly health education sessions. Patients were eligible if they were HIV-infected, on ART, were attending the BRHATC for any service (including counseling, clinical follow-up or drug refill), spoke English, and were willing to provide informed consent. Although more than 30 local languages exist, English is the language used for regular clinical care in the BRHATC and throughout the Northwest Region of Cameroon; more than 95% of clinic attendees can communicate effectively in English. Each participant could be eligible only once during the study period.

Our sampling goal was to recruit a study sample representative of HIV-infected patients receiving antiretroviral therapy at BRHATC. In the absence of a daily patient register to provide a sampling frame, study staff approached each patient consecutively as the patients passed through a central point in the registration process (weight measurement) until a patient indicated willingness to participate in the study. The recruiting staff member then obtained written informed consent from the interested patient and completed the first part of data collection. The patient would then complete the second part of data collection with a second staff member while the recruiting staff member resumed approaching patients consecutively at registration.

### Instruments

Lifetime and recent Major Depressive Disorder (MDD) were assessed using the World Health Organization’s Composite International Diagnostic Instrument (CIDI), a lay-administered diagnostic instrument whose performance has been validated widely in multiple international settings, [26], [27] including HIV settings. [6] The CIDI is a comprehensive, fully structured interview designed to be used by trained lay interviewers for the assessment of mental disorders in epidemiological and cross-cultural studies as well as for clinical and research purposes. We used the World Mental Health Survey Initiative Version of the CIDI (WMH-CIDI), which allows the assessment of mental disorders according to the definitions and criteria of ICD-10 and DSM-IV. [28], [29] The diagnostic section of the interview expands upon earlier versions of the CIDI by adding detailed questions about disorder severity, impairment, service use, and treatment, and has improved generalizability with increased involvement of less wealthy countries. [29], [30] This version has been successfully used in Sub-Saharan Africa (Nigeria). [29], [30] We administered the Screening and Depression modules of the WMH-CIDI. Interviews were conducted in person by a health care professional who received formal CIDI training from a WHO-certified trainer.

### Measures

Following standard CIDI scoring methodology, participants were assigned a diagnosis of a *lifetime episode of* MDD if they described ever experiencing a period meeting the following criteria: a period lasting at least two weeks characterized by at least 5 out of 9 core depressive symptoms, with at least one symptom being either depressed mood or anhedonia, and representing a change from previous functioning; the symptoms caused clinically significant distress or impairment in social, occupational, or other important areas of functioning; the symptoms were not better explained by substance use or a general medical condition; and the symptoms were not better explained by bereavement, or if secondary to bereavement, the episode either lasted more than two months or was characterized by at least one of the following: marked functional impairment, morbid preoccupation with worthlessness, suicidal ideation, psychotic symptoms, or psychomotor retardation.

On the CIDI, participants reporting any lifetime episode of MDD were asked if they had experienced any similar episodes in the past year, past 6 months, and past month. Participants reporting an episode in the past year or more recently completed a standard depressive severity rating scale called the Quick Inventory of Depressive Symptoms (QIDS) [31] that is embedded within the CIDI. The QIDS total score can range from 0–27 and has standard categories that correspond to very severe (21–27), severe (16–20), moderate (11–15), mild (6–10), and no (0–5) depressive symptoms. Participants were classified as having a diagnosis of a MDD in the past year, past 6 months, and past month if they endorsed a depressive episode in those time frames and received a score of 11 or above on the QIDS.

Participants additionally provided information on socio-demographics and on whether in the past 6 months they had experienced each of 13 symptoms commonly associated with HIV infection: [4] new or persistent headaches, fevers, oral pain, white patches in the mouth, rashes, nausea, trouble with eyes, sinus infection, numbness in the hands or feet, persistent cough, diarrhea, weight loss, or (for women only) abnormal vaginal discharge.

### Statistical Analysis

Participant characteristics, prevalence of depression, and characteristics of depressive episodes are described with proportions or medians and interquartile ranges (IQR). We describe continuous variables with medians and interquartile ranges due to the skewed distributions of some of these variables. Logistic regression was used to examine potential predictors of a diagnosis of depression in the past 6 months and in the past year. Odds ratios (OR) from bivariable analyses are used to describe the association of each predictor variable with depression. Age (divided by 10) and number of reported HIV symptoms were each modeled as simple linear terms after confirmation of the appropriateness of this assumption. Thus the OR for age represents the predicted increase in the odds of depression associated with a 10-year increase in age, and the OR for HIV symptoms represents the predicted increase in the odds of depression associated with each additional HIV symptom reported. For multivariable logistic regression analyses, the number of variables to be included in each of the models was decided *a priori* based on prevalence of the outcome; variables were selected for inclusion based on prior knowledge of relationship to the outcome and on relevance to the study question. All statistical analyses were carried out using Stata statistical software (version 11.1; STATA Corp, College Station, Texas).

## Results

### General Characteristics of Study Population

Of the 400 HIV-infected participants enrolled in the study, nearly ¾ were female and most were between 34 and 47 years of age (median 41 years) (Table 1). Most had previously been married (44%) or were currently married or cohabitating (34%). The majority (61%) had no more than a primary level of education (≤6 years). Participants reported a median of 5 (IQR: 3–6) physical symptoms commonly associated with HIV infection.

**Table 1:** Characteristics of the overall study population (N [%] or median [IQR]).

	Study population, n = 400	
	N/median	%/IQR
Sex		
Male	103	26
Female	297	74
Age	41	34–47
Religion		
Christian	394	99
Other	5	1
Marital Status		
Married/cohabitating	137	34
Previously married	178	44
Never married	85	21
Education*		
Primary	245	61
Greater than primary	155	39
Daily expenditures**	1	1–3
Village of residence		
Urban	244	61
Rural	156	39
Competency in English***		
Excellent	158	40
Fair	242	60
HIV symptom score (possible range: 0–13)	5	3–6

* Primary = 6 years or fewer; greater than primary = more than 6 years.

** In US dollars, approximation based on reported weekly expenditures in FCFC.

*** By interviewer assessment.

### Prevalence of MDD and Clinical Features

One in five participants met lifetime criteria for major depressive disorder (Table 2). The prevalence of MDD was 7% in the prior year, 5% in the prior 6 months, and 3% in the prior month.

**Table 2:** Prevalence of depression diagnoses (N, % or median, IQR).

	Study population, n = 400	
	N/median	%/IQR
Depression Diagnosis		
Past Month	11	3
Past 6 Months	20	5
Past Year	29	7
Lifetime	84	21
Number of lifetime episodes	2	1–3
Lifetime Diagnosis, n = 84		
Age at 1^st^ onset, years	34	25–40
Length of 1^st^ episode, days	122	30–1095
Ever talk to MD/other professional	28	33
Ever receive effective depression treatment	10	12
Past-year Diagnosis, n = 29		
QIDS score	16	14–18

For those who had experienced MDD in their lifetime, the median age at first onset of MDD was 34 years, and the median number of lifetime episodes was 2. The median length of the 1^st^ episode was 122 days (approximately 4 months). Only 1/3 had ever spoken with a physician or other health professional about depression, and 1/8 (12%) reported ever having received treatment that was helpful or effective for their depression.

### Characterizing the Most Recent Depressive Episode

**Figure 1:**
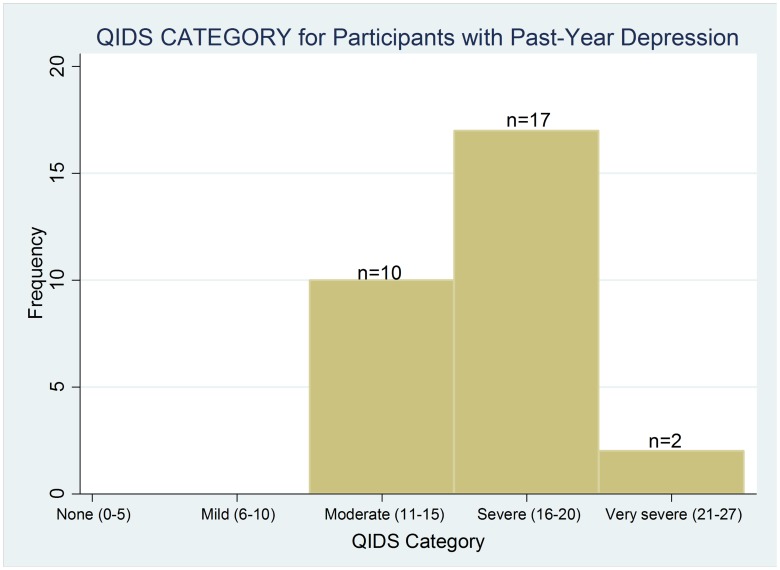
Categorized distribution of scores on the Quick Inventory of Depressive Symptomatology (QIDS) tool among participants with past-year depression.

Among those with MDD in the past year, the severity of the current episode (among those currently experiencing a major depressive episode) or the worst episode in the past year was pronounced, as reflected by a mean score of 16 on the QIDS instrument. As measured by the QIDS, 2/3 of patients depressed within the past year had either a severe (59%) or very severe (7%) episode (Figure 1).

We further delineated the specific depressive symptoms endorsed by depressed HIV-infected patients on the QIDS, as indicated by endorsing a symptom as present (Table 3). Among those with an episode in the past year, depressed mood and anhedonia were extremely common and nearly always presented together (97% of the sample reported each alone, and 100% had at least one of the two.) All depressed patients endorsed some type of insomnia (most commonly middle insomnia) and decreased attention/concentration. Nearly all endorsed feeling worthless or guilty and having a decreased level of energy (97% for each). Psychomotor retardation, weight change (nearly always weight loss), and appetite change (nearly always decreased appetite) were similarly frequently reported (93%, 93%, and 90% respectively). Restlessness and too much sleep were less frequently reported (73% and 59%, respectively). Thoughts of death or suicide were frequently endorsed by depressed HIV patients (86%).

**Table 3:** Depressive symptoms endorsed.

	Current episode/worst episode in last year (n = 29)	
	N	%
Feeling sad	28	97
Anhedonia	28	97
Any insomnia	29	100
Initial insomnia	26	90
Middle insomnia	29	100
Terminal insomnia	24	83
Decreased attention/concentration	29	100
Feeling worthless/guilty	28	97
Decreased level of energy	28	97
Psychomotor retardation	27	93
Weight changes	27	93
Weight gain	2	7
Weight loss	25	86
Appetite changes	27	90
Decreased appetite	26	90
Increased appetite	0	0
Thoughts of death/suicide	25	86
Restlessness	21	72
Too much sleep	17	59

For those patients who endorsed suicidal ideation, most reported having passive thoughts without active thoughts of harming themselves (Table 4). The great majority of those endorsing suicidal ideation reported thoughts that life was not worth living but had no thoughts of suicide, consistent with passive suicidal ideation (80%); 16% reported thoughts of suicide or death several times per week; and 4% endorsed plans or having recently made an attempt.

**Table 4:** Description of Suicidal Ideation.

	Depression Diagnosis in Past Year with Suicidal Ideation (n = 25)	
	N	%
Life empty/not worth living	20	80
Thoughts of suicide/death several times/week	4	16
Thoughts of suicide/death several times/day w/plans or attempt	1	4

### Correlates of Depression Diagnosis–bivariable Analysis

With bivariable analysis, we assessed whether common predictors were correlated with depressive diagnoses within the past year in this sample (Table 5). Females tended to be less likely than males to have depression, although this finding was not statistically significant. Older patients tended to be less likely to have MDD than younger ones. Those with education beyond the primary level tended to be twice as likely to have MDD as those with only primary education.

**Table 5:** Correlates of depression diagnosis – bivariable analysis.

	Depression Diagnosis in Past Year		
	OR	95% CI	
Gender			
Male	REF		
Female	0.63	0.28, 1.41	
Age*	0.82	0.50, 1.23	
Marital Status			
Married/cohabitating	REF		
Previously married	1.12	0.46, 2.70	
Never married	1.27	0.45, 3.54	
Education			
Primary	REF		
Greater than primary	2.04	0.95, 4.36	
Number of prior lifetime depressive episodes			
0	REF		
1	6.63	2.13, 20.59	
2+	12.14	4.51, 32.67	
HIV Symptoms**	1.19	1.05, 1.36	
Village of residence			
Urban	REF		
Rural	0.81	0.37, 1.80	

* Estimates for a 10-year increase in age.

** For each additional symptom.

Two variables stood out as significant predictors of MDD within the past year. First, the number of HIV symptoms was positively associated with the odds of having MDD within the past year. Each additional HIV symptom was associated with approximately a 20% increased odds of MDD. Second, prior history of MDD was the strongest risk factor for MDD within the past year. For those with a history of one episode of MDD more than a year ago, the likelihood of MDD within the past year increased nearly seven-fold compared to those without such a history, while a prior history of two or more episodes of MDD increased the likelihood of MDD within the past year by more than twelve-fold.

### Correlates of Depression Diagnosis–multivariable Analysis

Considering all these variables together in a multivariable analysis, findings remained consistent, with the number of prior depressive episodes and the number of HIV symptoms having significant associations with past-year MDD similar in magnitude to those found in bivariable analyses (Table 6). Female patients and patients living in rural areas tended to be less likely to have MDD within the preceding year.

**Table 6:** Correlates of depression diagnosis – multivariable analysis.

	Depression Diagnosis in Past Year		
	OR	95% CI	
Gender			
Male	REF		
Female	0.61	0.23, 1.64	
Age*	1.03	0.61, 1.73	
Marital Status			
Married/cohabitating	REF		
Previously married	1.57	0.53, 4.62	
Never married	1.24	0.36, 4.32	
Education			
Primary	REF		
Greater than primary	1.03	0.38, 2.81	
Number of prior lifetime depressive episodes			
0	REF		
1	5.42	1.65, 17.75	
2+	11.38	4.04, 32.04	
HIV Symptoms**	1.22	1.05, 1.42	
Village of residence			
Urban	REF		
Rural	0.72	0.29, 1.81	

* Estimates for a 10-year increase in age.

** For each additional symptom.

## Discussion

In this sample of HIV-infected patients receiving antiretroviral therapy and attending a regional AIDS treatment center in Cameroon, 3% met criteria for a major depressive episode within the past month, 5% had an episode within the past 6 months, 7% had an episode in the past year, and 21% met criteria for major depressive disorder at some point in their life. These prevalence values are consistent with prior reports on the frequency of MDD in African HIV-infected populations receiving antiretroviral therapy as determined by structured diagnostic interviews in which functional impairment is part of the diagnosis, such as the CIDI. Maj et al. reported that past month prevalence of MDD in Nairobi, Kenya was 3.0% and 5.5% in asymptomatic and symptomatic HIV-infected patients, respectively; rates in Kinshasa, Democratic Republic of the Congo, were 0 and 4.4%, respectively. [6] Current MDD was present in 2.7% of HIV-infected patients in a rural setting in Muheza, Tanzania. [32]

A small number of more recent studies have used as the diagnostic standard either the PRIME-MD mood module [33] or Mini International Neuropsychiatric Interview, [34], [35], [36], [37], [38] tools which do not consider functional impairment in diagnosing MDD. Accordingly, these studies may employ a more sensitive but less specific instrument to detect depression. These studies have reported a greater range of prevalence and higher rates of MDD, ranging from 8–34% in samples of HIV-infected individuals from Botswana, Nigeria, South Africa, and Uganda. [33], [34], [35], [36], [37], [38] In any case, our findings, coupled with those already reported in the literature, identify depression as an illness in Cameroon with a prevalence as high as that of other HIV-associated conditions, such as tuberculosis (0.041%) [39] and Hepatitis B virus (8.3%), [40] which have care recommendations incorporated into World Health Organization guidelines. [41]

Further, the lifetime MDD prevalence of 21% in this sample was 2–3 times higher than what has been reported in urban sub-Saharan African settings, suggesting that the burden of depression in HIV patients may be greater than expected based on studies in major African cities. Indeed, in Nairobi, Kenya, 6.1% of asymptomatic patients and 9.7% of symptomatic patients had a lifetime history of MDD, and in Kinshasa (Democratic Republic of Congo), 3.8% and 7.3%, respectively, had a lifetime history of MDD. [6]

Identification and successful management of MDD by a health care professional, a key objective of blue-ribbon panel reports and the World Health Organization, [24] was uncommon. Discussion of depression with clinicians was low, with only 1/3 reporting having spoken with a health professional about the depression, and only 1/8 reported every receiving helpful or effective depression treatment.

Key variables discriminating between HIV-infected patients with and without depression were the number of prior lifetime episodes and the number of physical symptoms potentially related to HIV. In adjusted analyses, having one prior episode of MDD increased the risk of having a recent MDD episode by over five-fold, while having two or more prior episodes increased the risk of recent MDD by over eleven-fold. We found no other reports on how prior history of MDD in HIV patients affected the risk of a recent MDD. For clinicians, then, a key clinical piece of information in evaluating current depression is whether a patient has a history of depressive episodes.

Similarly, having a greater number of HIV symptoms increased the odds of having MDD within the past year. This finding is consistent with reports of prior studies suggesting an association between depression and severity of HIV illness. [42], [43]

Depressive symptomatology in this population mirrored presentations of depression in other settings, and was notable for infrequent reports of weight gain or increased appetite. Middle insomnia, experienced by all depressed HIV patients in this sample, is a symptom that HIV clinicians might be able to address quickly and effectively with amitriptyline, the most commonly used and widely available antidepressant in Cameroon.

Suicidal ideation was common in depressed HIV patients (86%), but it was primarily passive, described as thoughts that life was not worth living rather than having active thoughts of self-harm. This high frequency of suicidal ideation in general with a low frequency of specifically active suicidal ideation is consistent with prior studies of depression in medical settings. [44]

Finally, the majority of individuals with MDD experienced severe rather than moderate depressive symptoms and were substantially impaired. Indeed, 2/3 met criteria for being severely or very severely depressed, which is a greater proportion than was reported in a rural sample in Tanzania (2/3 moderately depressed, 1/3 severely depressed). [32]

Our findings are limited by a number of issues. First, while consistent with prior reports, our prevalence of current depression was still smaller than we expected, limiting our ability to comprehensively assess variables predictive of major depressive disorder. We used a well validated and culturally translated instrument, and depression diagnoses were carefully reviewed by a psychiatrist, so the measurement of depression is likely to have high construct validity. The large majority of cases of depression were severe or very severe, so the predictors identified are likely to be good predictors of severe, clinically relevant depressive disorders with substantial impairment. Second, following our diagnostic reference standard, the CIDI, we collected a full report on depressive symptoms only on patients with MDD, so we could not identify depressive symptoms that distinguish between clinically depressed HIV patients and HIV patients with mild depressive symptoms who are not clinically depressed. Such information would be helpful to HIV clinicians to focus on key depressive symptoms that might differentiate those at higher risk of clinical depression. Third, these data are from a single site in a small urban center in Cameroon, which may limit their generalizability. However, findings here were generally consistent with what has been reported in other sub-Saharan Africa sites. Fourth, in the assessment of predictors of MDD, there may be some uncontrolled confounding because of the small number of variables that could be included in the multivariate analysis. Finally, most of the variables and measurements were self-reported, so their accuracy is dependent on the veracity of the participants.

### Conclusions

This study of HIV-positive outpatients attending a hospital in a small urban center in Bamenda, Cameroon, found that 7% had depression at some point within the past year, most of which was severe or very severe. Identification and successful management of MDD by a health care professional was infrequent. Suicidal ideation was common, although usually passive. Important predictors of a major depressive episode in the past year were prior history of depression and a greater number of HIV symptoms. Future research can look at interventions to help HIV clinicians better identify and manage this common and complicating comorbidity. The management of depression (including screening and appropriate care) needs to be incorporated in HIV-care guidelines in Cameroon and other similar settings, as its prevalence is as high as those of other HIV-associated conditions such as tuberculosis and hepatitis B infection whose management is included in such guidelines.
